# Swallowing abnormalities in HIV infected children: an important cause of morbidity

**DOI:** 10.1186/1471-2431-12-68

**Published:** 2012-06-14

**Authors:** Etienne D Nel, Alida Ellis

**Affiliations:** 1 Department of Paediatrics and Child Health, Stellenbosch University and Tygerberg Academic Hospital, Cape Town, South Africa; 2 Speech and Hearing Department, Stellenbosch University and Tygerberg Academic Hospital, Cape Town, South Africa

## Abstract

**Background:**

Swallowing disorders, well recognised in adults, contribute to HIV-infection morbidity. Little data however is available for HIV-infected children. The purpose of this study is to describe swallowing disorders in a group of HIV-infected children in Africa after the introduction of combined anti-retroviral therapy.

**Methods:**

We describe 25 HIV-infected children referred for possible swallowing disorders. Clinical and videofluoroscopic assessment of swallowing (VFSS), HIV stage, and respiratory and neurological examination were recorded.

**Results:**

Median age was 8 months (range 2.8-92) and 15 (60%) were male. Fifteen (60%) were referred for recurrent respiratory complaints, 4 (16%) for poor growth, 4 (16%) for poor feeding and 2 (8%) patients for respiratory complaints and either poor growth or feeding. Twenty patients (80%) had clinical evidence of swallowing abnormalities: 11 (44%) in the oral phase, 4 (16%) in the pharyngeal phase, and 5 (25%) in both the oral and pharyngeal phases. Thirteen patients had a videofluoroscopic assessment of which 6 (46%) where abnormal. Abnormalities were detected in the oral phase in 2, in the pharyngeal phase in 3, and in the oral and pharyngeal phase in 1; all of these patients also had evidence of respiratory involvement. Abnormal swallowing occurred in 85% of children with central nervous system disease. CNS disease was due to HIV encephalopathy (8) and miscellaneous central nervous system diseases (5). Three of 4 (75%) patients with thrush had an abnormal oral phase on assessment. No abnormalities of the oesophagus were found.

**Conclusions:**

This report highlights the importance of swallowing disorders in HIV infected children. Most patients have functional rather than structural or mucosal abnormalities. VFSS makes an important contribution to the diagnosis and management of these patients.

## Background

Swallowing disorders, often unrecognized in AIDS and other chronic diseases, significantly reduce quality of life [1] and increase morbidity and mortality: nutrition is adversely affected [2], the ability to take medication is reduced [3] and serious complications such as aspiration pneumonia [4] may occur. The causes of swallowing disorders are multi-factorial and include infections of the mouth, pharynx and esophagus, functional abnormalities of swallowing, and structural abnormalities of the oral cavity, pharynx and esophagus. Early diagnosis and treatment of these disorders may improve the outcome of HIV infected patients [5].

A wide range of swallowing difficulties occurs in HIV infected adults. Almost 80% of adults in an HIV clinic in South Africa [1] reported symptoms of swallowing disorders that adversely affected quality of life. Odynophagia and difficulty swallowing specific foods were common: the oral, pharyngeal, and oesophageal phases of swallowing were involved. Although patients with lower CD4 counts had more severe symptoms, even patients who were not severely immune compromised often had swallowing abnormalities.

Videofluoroscopic studies of HIV infected adults [4] with dysphagia show abnormalities in the oral and pharyngeal phases of swallowing, often in the absence of signs of oesophagitis or local pathology of the mouth or pharynx. Aspiration, that is often silent, is common and may lead to pneumonia.

The limited pediatric data that is available precedes the introduction of combination anti-retroviral therapy (cART). Forty-five percent of children with AIDS in a case-series preceding the introduction of cART had dysphagia [5]. Of these, 24% had thrush, 48% had developmental delay, and 12% had progressive encephalopathy. Most patients had improved oral intake and weight gain with manipulations such as postural changes during feeds and dietary changes. However, little is known about swallowing disorders after the introduction of combined anti-retroviral treatment (cART) or in HIV-infected children in Africa.

We describe our experience with HIV-infected children referred for assessment of swallowing at Tygerberg Children’s Hospital in Cape Town, South Africa, a referral unit in a high HIV prevalence area. It is estimated that 16% of women attending antenatal clinics in the Western Cape Province in South Africa are HIV infected [6] and that 1.1% of children between 2 and 14 years are HIV infected [7].

## Methods

Twenty-five HIV infected children were referred for suspected swallowing disorders from June 2006 through March 2009. Clinical details (HIV stage, presence of neurological disease, HIV encephalopathy, lung disease) were obtained from patient records.

Clinical assessment of feeding, as described by Logemann [8,9], was performed. This assessment includes examination of oral anatomy for malformations or muscle weakness, of posture and the presence of reflexes of swallowing, coughing or gagging. The speech and language therapist also observes the patient for signs of aspiration during feeding and swallowing. Patients were referred for a videofluoroscopic swallow study (VFSS) if a pharyngeal swallowing abnormality or aspiration were suspected.

Standard videofluoroscopy equipment was used. Children were positioned supine or standing depending on age and motor skills. Every child received a routine upper gastrointestinal series prior to the videofluoroscopy to assess the upper gastrointestinal tract. Children were given Omnipague® first to assess for aspiration before thin barium was given. Thin barium (diluted with water) was given via syringe or bottle depending on the feeding skills of the child. In children older than 3 months, thickened barium was also presented as a puree or syrup consistency with a spoon.

After the videofluoroscopy, the radiologist and speech and language therapist commented on the oral and pharyngeal phases, timing of swallow, and aspiration (silent or symptomatic). Specific strategies to promote oral feeding and other long-term feeding options were suggested.

HIV infection was diagnosed by HIV RNA PCR in children below age 15 months and HIV-specific antibodies by ELISA in children over 15 months. CD4 counts are expressed as absolute counts and percentages. Clinical and immunological staging are according to the World Health Organisation (WHO) guidelines for immunological and clinical classification for children with confirmed HIV infection (2006) [10].

### Statistical analysis

Results are summarized as medians with ranges. A Chi-squared test was used to compare categorical variables. Where the expected value of one of more cells was less than 5, the Fisher exact test was used. Medians were compared with the Mann- Whitney *U* Test. Statistical analysis was performed with Statistica® version 9 (Statsoft Inc.).

This study was carried out in compliance to the Helsinki Declaration and permission to conduct the study was granted by the Human Ethics Research Committee of the Faculty of Health Sciences of Stellenbosch University.

## Results

The median age of the 25 children was 8 months (range 2.8-92) and 15 (60%) were male. Fifteen (60%) were referred for recurrent respiratory complaints, 3 (12%) for poor growth, 4 (16%) for poor feeding and 2 (8%) patients for respiratory complaints and poor growth and 1 (4%) for poor feeding and respiratory complaints. No specific swallowing disorder was identified in any of the patients prior to referral.

Twenty patients (80%) had clinical evidence of swallowing abnormalities: 11 (44%) in the oral phase, 4 (16%) in the pharyngeal phase and 5 (25%) in both the oral and pharyngeal phases (Table 1).

**Table 1:** Clinical features of patients with results of assessment

		**Clinical assessment**			**VFSS**		
**Indication for assessment**	**Swallow abnormality**	**Oral**	**Pharyngeal**	**Both**	**Oral**	**Pharyngeal**	**Both**
Recurrent respiratory infections (n = 18)*	15 (83%)	6	4	5	1	3	1
Poor feeding (n = 5)	5	5	0	0	2	0	0
Growth failure (n = 5)	2 (40%)	1	1	0	0	0	0

* Two patients also had poor growth and 1 poor feeding.

Thirteen patients had a VFSS of which 6 (46%) were abnormal; all of these also had an abnormal clinical assessment. Abnormalities were detected in the oral phase in 2, in the pharyngeal phase in 3, and in the oral and pharyngeal phase in 1 (Table 1).

Six (24%) patients aspirated during assessment: 4 patients during clinical assessment (2 of these also had radiological evidence of aspiration); 1 patient aspirated during VFSS without symptoms of aspiration (silent aspiration); and 1 patient aspirated after an episode of gastroesophageal reflux. Five of the 6 (83%) patients with aspiration had been referred for recurrent respiratory infections and the other was subsequently found to have radiological evidence of lung disease.

Sixteen patients were receiving cART at the time of initial assessment for a median of 55 days (Range 11–392 days). Thirteen of these (81%) had swallow disorders in comparison to 7 of 9 (78%) patients who were not yet on cART (NS). There was no difference in the CD4 counts (expressed as a percentage of total T-cells and as an absolute count) between those patients on and off cART.

Three of four (75%) patients with stage 3 clinical disease had abnormal swallowing and 16 of 20 (80%) with Stage 4. CD4 Counts were available for 19 (76%) patients within 60 days of clinical assessment or VFSS; swallow abnormalities were present in 10 of 13 (77%) severely immune-suppressed children, and all children with advanced, mild or no immune suppression; this difference was not statistically significant (p = 0.52). There was also no difference in absolute CD4 counts and CD4 counts expressed as percentages of T lymphocytes between children with and without swallowing disorders (Table 2).

**Table 2:** Presence of swallowing disorders by clinical and immunological staging of patients

	**Swallowing disorder present**	**No swallowing disorder**	**Total**	***p***
**Clinical stage (n = 25)**	Stage 3: 3	Stage 3:1	4 Stage 3;	NS
	Stage 4: 17	Stage 4: 4	21 Stage 4	
**Immunological stage (n = 19)**	None: 3	None: 0	None 3; Mild 2;	NS
	Mild: 2	Mild: 0	Advanced 1;	
	Advanced: 1	Advanced: 0	Severe 13	
	Severe: 10	Severe: 3		
**Absolute CD4 count**	760 x10^9^/l	1364x10^9^/l	778 x10^9^/l	0.08
	(7-4573)	(1191-1746)	(7-4573) (n = 19)	
	(n = 16)	(n = 3)		
**Cd4 count percentage**	19.5%	18%	19%	NS
	(5-38)	(18-23)	(5-38)	
	(n = 16)	(n = 3)	(n = 19)	

Thirteen of 25 patients had central nervous system disease: 8 with HIV encephalopathy and 5 with miscellaneous central nervous system diseases (cerebral palsy, seizures after meningitis, developmental delay). Of these, 11 (85%) had abnormal swallowing: 9 in the oral phase and 7 in the pharyngeal phase (5 patients have abnormalities in both the oral and pharyngeal phases). Patients with neurological abnormalities were not more likely to have swallowing abnormalities than those without (p = 0.64).

Three of 4 (75%) patients with thrush had an abnormal oral phase: 2 with clinical assessment only, and 1 with clinical assessment and VFSS. One patient also had symptoms of aspiration. No anatomic abnormalities of the oesophagus were demonstrated with contrast studies.

## Discussion

This case-series highlights the importance of assessing HIV-infected children for swallowing disorders, in particular those with recurrent respiratory tract infections and neurological disease.

The spectrum of patients described reflects the referral pattern to our swallowing disorder clinic. The most common indication for referral was recurrent lung infections. The majority of these patients (88%) had abnormal swallowing and a third of them aspirated during assessment. This is similar to the finding of Halvorsen [4] that almost half of adults assessed with VFSS aspirated. Clinical assessment underestimates the true number of patients in whom aspiration occurs. In our study, VFSS identified 2 patients with aspiration in whom it was not suspected clinically and significantly changed their management. The importance of silent aspiration in the development of lung disease in HIV infected children is unknown, it should however be considered as a possible contributing factor in those with chronic or recurrent lung disease of uncertain etiology [11,12].

Another explanation for the high prevalence of swallowing abnormalities in children referred with recurrent lung infections is that both lung infections and swallowing disorders are more likely to occur in patients with advanced HIV infection. Bladon [1] found that patients with low CD4 counts were more likely to have swallowing disorders. There was however no difference in the CD4 counts or clinical stage of HIV infection of those patients with or without swallow disorders in our series. This may be because patients with swallowing disorders were more likely to be referred for assessment and the small number of patients included. The relatively short duration of treatment prior to assessment may explain the apparent lack of effect of cART on the CD4 count. The short duration of cART in most patients was possibly also too short to make a clinical difference. While improvements in immunological status, weight gain, and well-being are observed within the first weeks of treatment, improvement in neurological and intestinal disease are often delayed.

Dysphagia is common in children with severe neurological disease [13-16]. The characteristics of swallowing disorders found in these children are similar to those found in non-HIV infected children with neurological impairment. Although most of the children with neurological disease in this series had some swallowing abnormality, this was not significantly more common than in children without signs of neurological disease.

Most swallowing abnormalities were due to functional disturbances of swallowing. These findings are consistent with studies in adults that showed abnormalities in all phases of swallowing in the absence of local pathology [4]. It should however be noted that esophagoscopy was not performed on any of these patients.

We describe all HIV infected referred for feeding and swallowing assessment over a period of 33 months. The HIV clinic in the hospital manages approximately 400 children with HIV infection. However, the present study does not allow us to estimate the prevalence of swallowing disorders in HIV infected children in our service. The spectrum of patients referred is also subject to a strong referral bias as children with respiratory disease and neurological abnormalities were more likely to be referred for evaluation. This bias may also account for the absence of any association between immunological and clinical stage of HIV infection and the presence of swallowing disorders.

## Conclusions

In this report of we bring attention to the importance of swallowing disorders in HIV infected children. In particular, children with recurrent lung infections often had swallowing disorders. Most patients had functional abnormalities rather than structural or mucosal abnormalities. VFSS makes an important contribution to the diagnosis and management of these patients.

## Competing interests

The author(s) declare that they have no competing interests'.

## Authors’ contributions

EDN is a paediatric gastroenterologist, carried out the data analysis and drafted the manuscript. AE is a speech therapist who performed the clinical and radiological assessment of swallowing. Both authors read and approved the final manuscript.

## Pre-publication history

The pre-publication history for this paper can be accessed here:

http://www.biomedcentral.com/1471-2431/12/68/prepub
